# The genome sequence of *Molossus alvarezi *González-Ruiz, Ramírez-Pulido and Arroyo-Cabrales, 2011 (Chiroptera, Molossidae)

**DOI:** 10.12688/wellcomeopenres.22726.1

**Published:** 2024-09-12

**Authors:** Nancy B. Simmons, Melissa R. Ingala, Myrtani Pieri, Brian P. O'Toole, Jonathan L. Gray, Philip Philge, Thomas L Volkert, Ning Zhang, Linelle Abueg, Nadolina Brajuka, Erich Jarvis, Giulio Formenti, Kirsty McCaffrey, Meike Mai, Emma C. Teeling, Sonja C Vernes, The Bat Biology Foundation, The Bat1K Consortium

**Affiliations:** 1 Department of Mammalogy, Division of Vertebrate Zoology, American Museum of Natural History, New York, NY 10024, USA; 2 Fairleigh Dickinson University Department of Biological Sciences, Madison, NJ, 07940, USA; 3 Department of Life Sciences, School of Life and Health Sciences, University of Nicosia, Nicosia, Cyprus; 4 Paratus Sciences, New York, USA; 5 Vertebrate Genome Lab, The Rockefeller University, New York, USA; 6 University of St Andrews School of Biology, St Andrews, Scotland, UK; 7 University College Dublin School of Biology and Environmental Science, Dublin, Ireland; 8 Wellcome Sanger Institute, Wellcome Genome Campus, Cambridgeshire, CB10 1SA, UK; 9 Neurogenetics of Vocal Communication Group, Max Planck Institute for Psycholinguistics, Nijmegen, The Netherlands

**Keywords:** Molossus alvarezi, genome sequence, chromosomal, Bat1K

## Abstract

We present a genome assembly from an individual female *Molossus alvarezi* (Chordata; Mammalia; Chiroptera; Molossidae). The genome sequence is 2.490 Gb in span. The majority of the assembly is scaffolded into 24 chromosomal pseudomolecules, with the X sex chromosomes assembled.

## Species taxonomy

Eukaryota; Metazoa; Eumetazoa; Bilateria; Deuterostomia; Chordata; Craniata; Vertebrata; Gnathostomata; Teleostomi; Euteleostomi; Sarcopterygii; Dipnotetrapodomorpha; Tetrapoda; Amniota; Mammalia; Theria; Eutheria; Boreoeutheria; Laurasiatheria; Chiroptera; Yangochiroptera; Molossidae; Molossinae; *Molossus*; *Molossus alvarezi ( Loureiro *et al.*, 2019; Loureiro *et al.*, 2020; Meredith *et al.*, 2011; Simmons & Cirranello, 2024; Teeling *et al.*, 2005)*.

## Introduction

**Figure 1.**
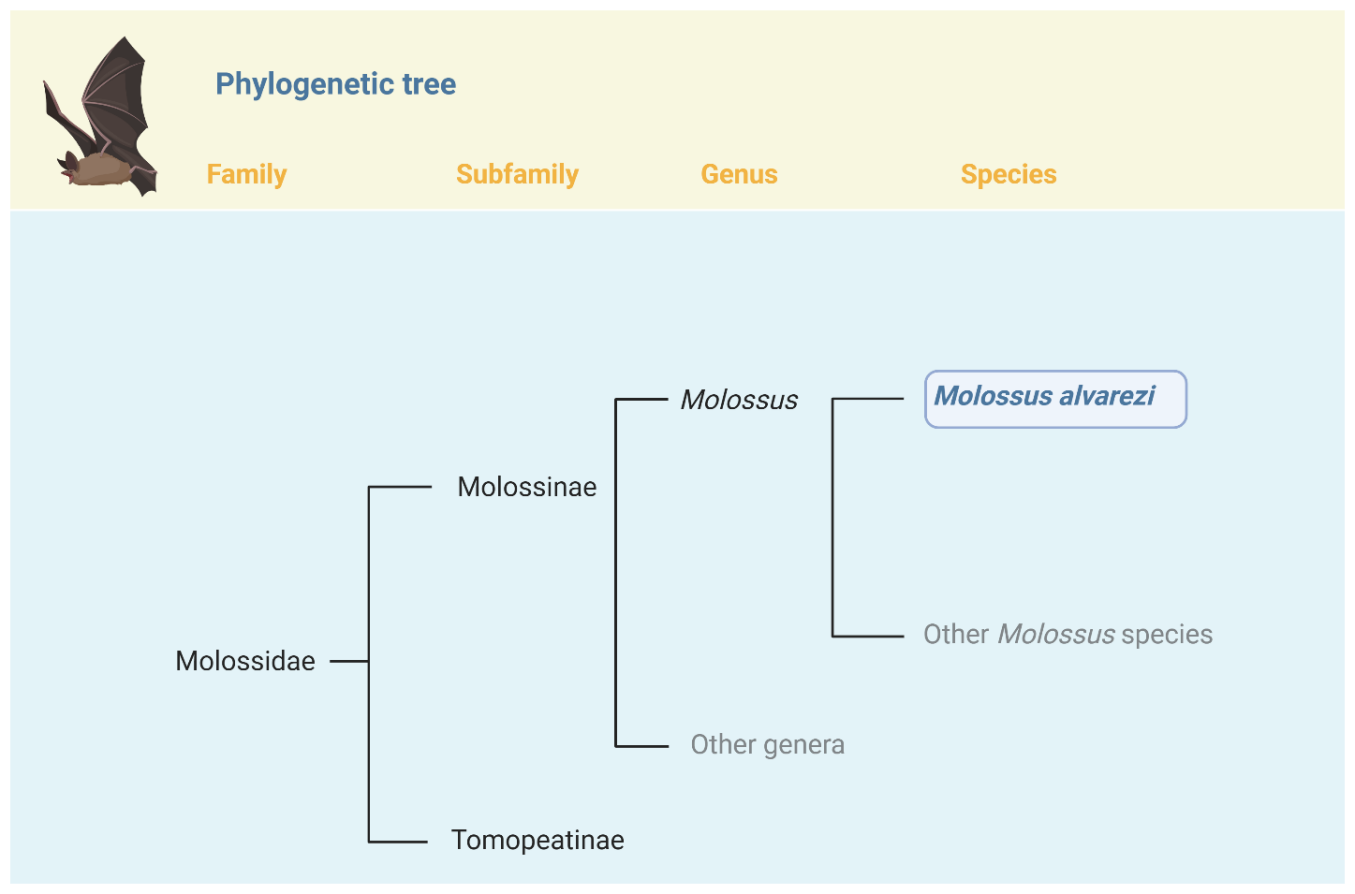
Position of *Molossus alvarezi* in the phylogeny of Family Molossidae. *Molossus alvarezi* is one of 15 species currently recognized in the genus *Molossus ( Loureiro *et al.*, 2019; Loureiro *et al.*, 2020; Simmons & Cirranello, 2024)*. *Molossus* belongs to the Subfamily Molossinae, which currently includes 20 genera and 133 species ( Simmons & Cirranello, 2024). Within *Molossus*, *M. alverezi* occupies a basal branch and is sister to a large clade of other species from Central and South America ( Loureiro *et al.*, 2020). Figure created with Biorender.com.

Molossid bats are swift aerial insectivores that are distributed throughout the world. As shown in Figure 1, they comprise two subfamilies, the South American endemic Tomopeatinae and the cosmopolitan Molossinae ( Eger, 2008b), the latter consisting of 21 genera and 131 species ( Simmons & Cirranello, 2024). Within this group, the genus *Molossus* comprises 15 species distributed broadly across the Neotropics ( Loureiro *et al.*, 2019; Simmons & Cirranello, 2024). *Molossus alvarezi* was described for the first time in 2011 by Gonzalez-Ruiz *et al.* ( González-Ruiz *et al.*, 2011) based on specimens from the Yucatán Peninsula of Mexico, but this species is now known to range from the Yucatán through Belize, Guatemala, and Honduras south to Colombia, Venezuela, Trinidad, Surinam, French Guiana, and Peru ( Simmons & Cirranello, 2024).

**Figure 2.**
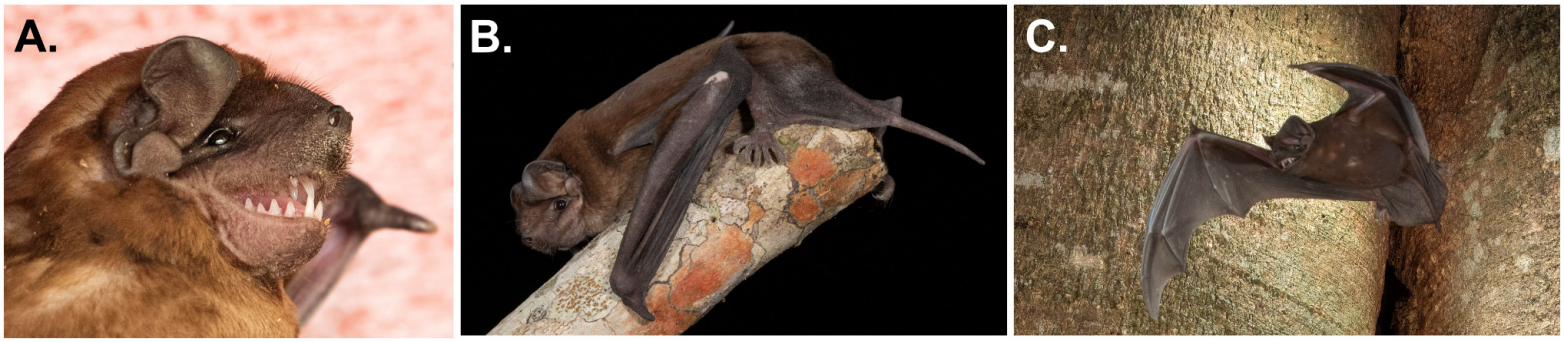
*Molossus alvarezi*. Adult individuals of *Molossus alvarezi* from Belize [Photo **A** taken by Brock and Sherri Fenton and photos **B** and **C** taken by Charles Francis].

*Molossus alvarezi* as shown in Figure 2 is a medium-sized molossid that is most morphologically similar to *Molossus sinaloae ( Eger, 2008a),* with which it was previously synonymized, although the two species do not appear to be particularly close relatives based on recent phylogenies ( Loureiro *et al.*, 2020). Forearm length of *M. alvarezi* varies from 42-48 mm; dorsal fur is chocolate brown while the venter is slightly paler and grayish ( González-Ruiz *et al.*, 2011; Miller, 2003). A key feature for distinguishing these bats in the field is that dorsal fur of *M*. *alvarezi* is bicolored with a white base that extends more than half the hair length ( González-Ruiz *et al.*, 2011; Miller, 2003). Other craniodental features also distinguish this species from congeners ( González-Ruiz *et al.*, 2011).

The diet, behavior, and echolocation calls of *M*. *alvarezi* are not known, although we presume that this species is an aerial insectivore that uses FM echolocation calls like other molossid bats ( Jung *et al.*, 2014). The species *M*. *alvarezi* is currently classified as Data Deficient in the IUCN Red List of Threatened Species ( Barquez *et al.*, 2015).

## Genome sequence report

The genome was sequenced from a single female *M. alvarezi* (field number BZ-4, catalog number AMNH:Mammalogy:280599) collected at Lamanai Outpost Lodge, Orange Walk District, Belize (17.75156 N, 88.65376 W) on 8 November 2021. A total of 51x-fold coverage in Pacific Biosciences Hi-Fi long reads (contig N50 63 Mb) was generated after removal of all reads shorter than 10kb. Primary assembly contigs were scaffolded with chromosome confirmation Hi-C data. The final assembly has a total length of 2.48 Gb in 504 sequence scaffolds with a scaffold N50 of 112.65 Mb ( Table 1). The assembly has a BUSCO ( Simao *et al.*, 2015) completeness of [98.2]% using the Laurasiatheria reference set. The assembly was fully phased and both haplotypes are deposited. Chromosomal pseudomolecules in the genome assembly of *M. alvarezi* are shown in Table 2.

**Table 1.** Genome data for *Molossus alvarezi*.

Project accession data	
Assembly identifier	GCA_037157525.1
Species	*Molossus alvarezi*
Specimen	mMolAlv2
NCBI taxonomy ID	1552295
BioProject	Accession: PRJNA944206; Bat1K: Accession: PRJNA489245; ID: 489245
BioSample ID	SAMN31836526
Isolate information	Female - Muscle
Genome assembly	
Assembly accession	GCA_037157525.1
Accession of Alternative haplotype	GCA_037157525.1 (Primary), GCA_037176705.1 (Alternative)
Span (Mb)	2,395.14
Number of contigs	1,369
Contig N50 length (Mb)	4,060.33
Number of scaffolds	187
Scaffold N50 length (Mb)	113,916.77
Longest scaffold (Mb)	270,994.32

* BUSCO scores based on the laurasiatheria_odb10 set using v5.0.0. C= complete [S= single copy, D=duplicated], F=fragmented, M=missing, n=number of orthologues in comparison. * *Molossus alvarezi* BUSCO scores based on laurasiatheria_odb10 BUSCO set v5.3.2.

**Table 2.** Chromosomal pseudomolecules in the genome assembly of *Molossus alvarezi*. ENA accession Chromosome Size (Mb) GC%. The chromosome number of *Molossus alvarezi* is 2n=24.

ENA accession	Chromosome	Size (Mb)	GC%
SUPER_1	1	278.8	0.4081
SUPER_X	2	150.3	0.3943
SUPER_2	3	134.3	0.4173
SUPER_3	4	131.9	0.4318
SUPER_4	5	120.3	0.4204
SUPER_5	6	120.1	0.4252
SUPER_6	7	119.8	0.4041
SUPER_7	8	116.9	0.409
SUPER_8	9	112.7	0.4059
SUPER_9	10	112.3	0.3984
SUPER_10	11	110.2	0.4077
SUPER_11	12	108.7	0.4366
SUPER_12	13	103.9	0.4288
SUPER_13	14	99.1	0.431
SUPER_14	X	81.9	0.4308
SUPER_15	15	77.4	0.4153
SUPER_16	16	74.9	0.4378
SUPER_17	17	73.5	0.4595
SUPER_18	18	69.7	0.4397
SUPER_19	19	61.1	0.4462
SUPER_20	20	58.6	0.4645
SUPER_21	21	39.4	0.4726
SUPER_22	22	31.5	0.4538
SUPER_23	23	17.1	0.4738

## Methods

The *M. alvarezi* specimen was a female individual captured on an American Museum of Natural History (AMNH) field expedition to the Lamanai area in the Orange Walk District of Belize. The bat was caught in a ground-level mist net set in the gardens at Lamanai Outpost Lodge (17.75156 N, 88.65376 W). All efforts were made to minimize any distress or suffering by the animal. The individual sampled was subjected to minimal handling after capture, and it was held in a clean cloth bag after capture as per best practices for field containment of bats ( Kunz & Parsons, 2009). After species identification, the individual was euthanized humanely by trained personnel the same night it was captured. Capture and sampling were conducted under Belize Forest Department Permit FD/WL/1/21, and samples were exported under Belize Forest Department permit FD/WL/7/22(08). The individual sampled was identified as *M. alvarezi* based on morphological traits and measurements described by Gonzalez-Ruiz *et al.* ( González-Ruiz *et al.*, 2011).

The animal was euthanized by isoflurane inhalation (<1 ml to moisten cotton ball), a humane approved method that rapidly causes unconsciousness and eventually death upon inhalation. Bats euthanized by this method are rendered unconscious within seconds due to their high respiration rate, and death occurs within a minute or two with no significant suffering by the animal. Tissues were removed from the subject individual immediately following euthanasia and were flash-frozen in a liquid nitrogen dry shipper, with the cold chain maintained from field to museum to laboratory. All data were recorded and reported in accordance with the ARRIVE guidelines – see data availability section and Table 1. All work was conducted with approval by the AMNH Institutional Animal Care and Use Committee (AMNHIACUC-20191212).

DNA was extracted using Nanobind extraction from muscle tissue following the Circulomics Nanobind HMW DNA Extraction Protocol. Pacific Biosciences HiFi libraries were constructed according to the manufacturer's instructions. Hi-C data was generated using the Arima Hi-C+ High Coverage kit from the same muscle tissue sample. Sequencing was performed by the Genomic Operations DNA Pipelines at Paratus Sciences on Pacific Biosciences Sequel IIe (HiFi reads) and Illumina NextSeq 2000 (Hi-C) instruments.

**Figure 3.**
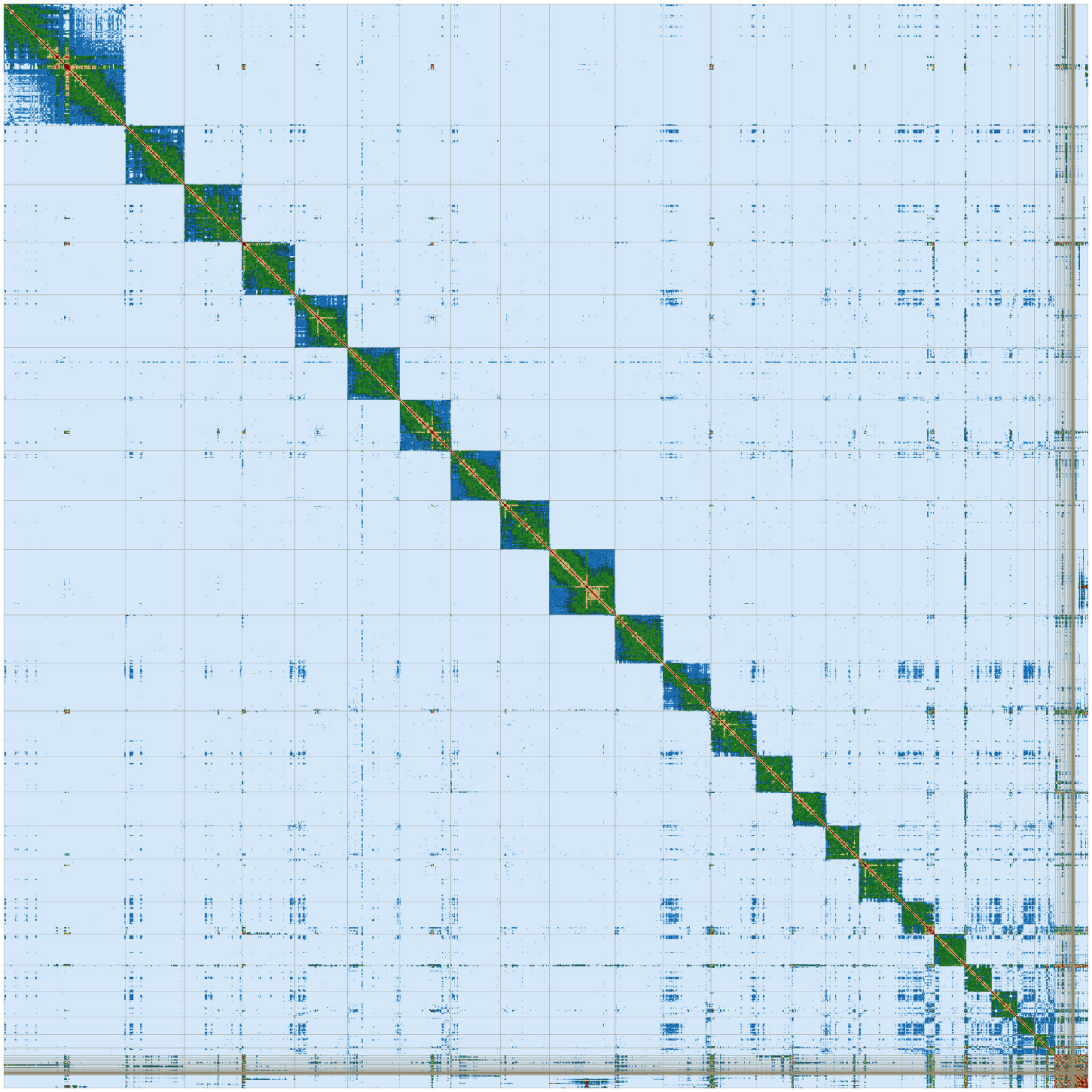
Hi-C Contact Map of the *Molossus alvarezi* assembly with 24 chromosomes, visualized using PretextView.

**Figure 4.**
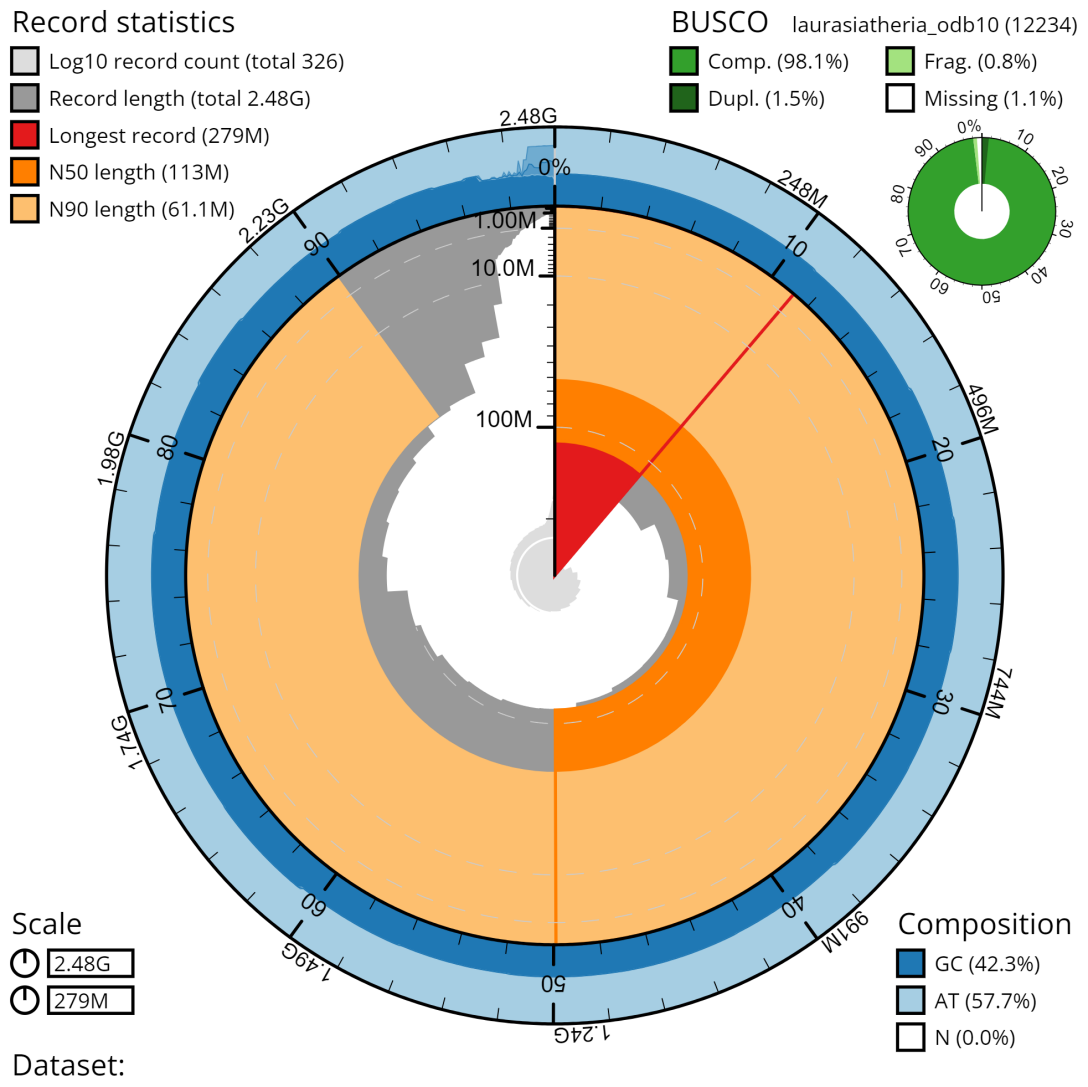
Genome assembly metrics generated using blobtoolkit for the *Molossus alvarezi* genome assembly. The larger snail plot depicts scaffold statistics including N50 length (bright orange) and base composition (blue). The smaller plot shows BUSCO completeness in green.

Assembly was carried out following the Vertebrate Genome Project Galaxy pipeline v2.0 ( Larivière *et al.*, 2024). A brief synopsis of the method is as follows: Genome size was estimated using GenomeScope2 ( Ranallo-Benavidez *et al.*, 2020). Hifiasm with Hi-C phasing was used for genome assembly ( Cheng *et al.*, 2022). The quality of the assembly was evaluated using Merqury ( Nurk *et al.*, 2020) and BUSCO ( Manni *et al.*, 2021). Scaffolding with Hi-C data ( Rao *et al.*, 2014) was carried out with YaHS ( Zhou *et al.*, 2023). PretextView was implemented to generate a Hi-C contact map ( Figure 3). Figure 4– Figure 6 were generated using BlobToolKit ( Challis *et al.*, 2020). All bioinformatics software utilised for the *M. alvarezi* analysis are depicted in Table 3.

**Figure 5.**
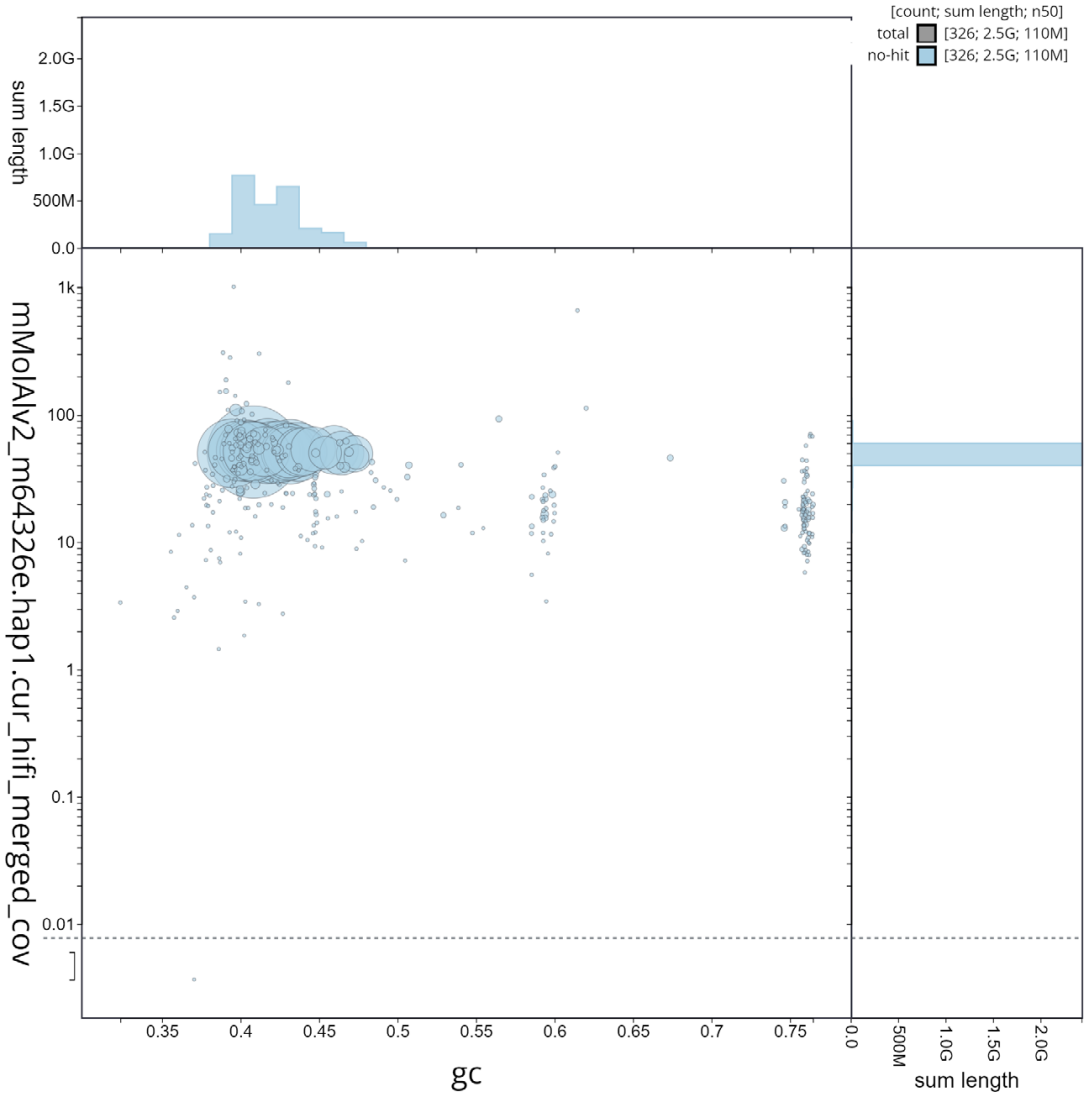
GC coverage plot generated for the *Molossus alvarezi* assembly using blobtoolkit. Individual chromosomes and scaffolds are represented by each circle. The circles are sized in proportion to chromosome/scaffold length. Histograms show the sum length of chromosome/scaffold size along each axis. Color of circles indicate taxonomic hits of each Phylum represented in the assembly.

**Figure 6.**
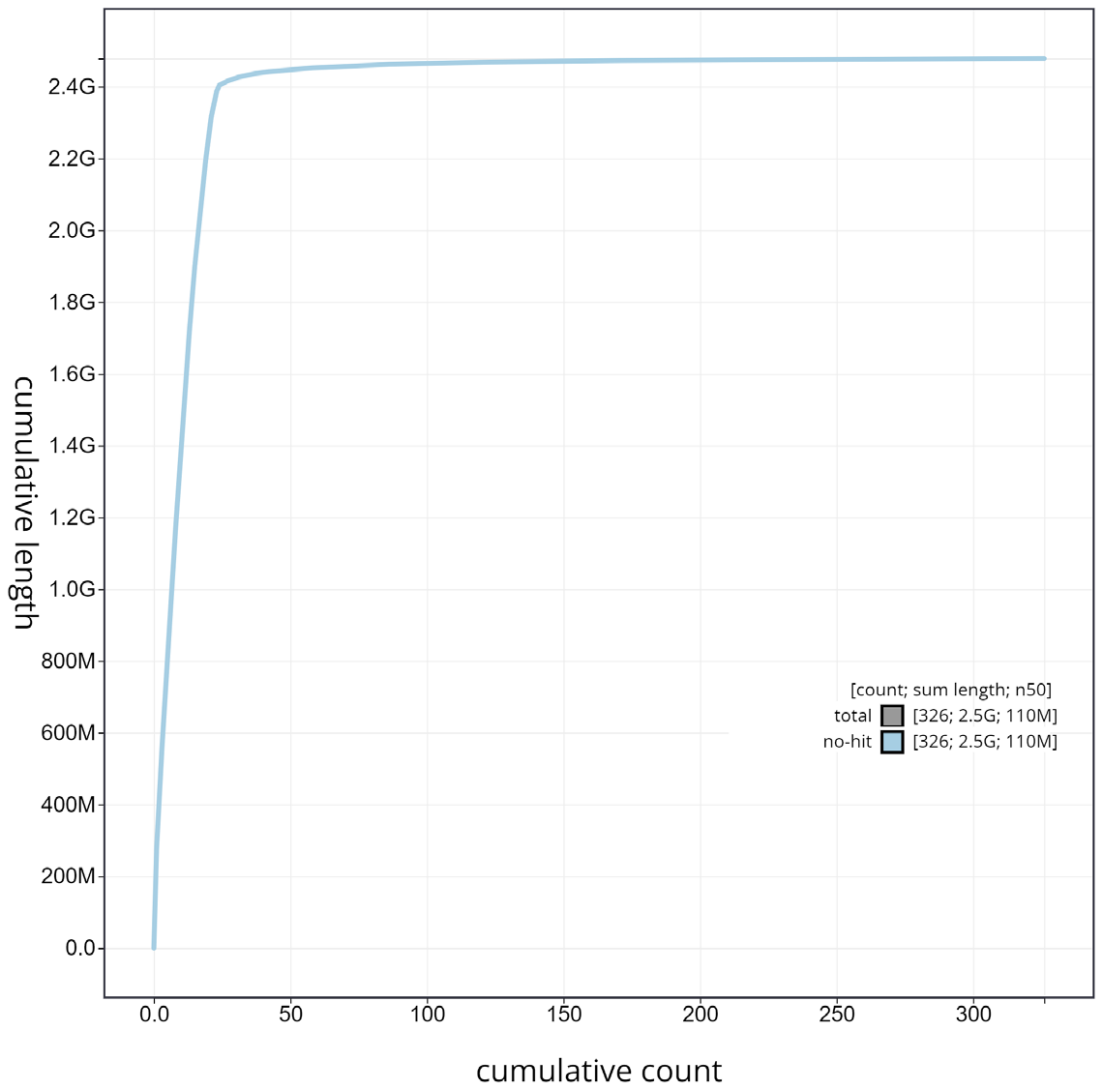
Cumulative sequence plot generated for the *Molossus alvarezi* assembly using blobtoolkit. The grey line shows the cumulative length for all chromosomes/scaffolds in the assembly. Colored lines represent Phylum represented in the assembly.

**Table 3.** Software tools used.

Software tool	Version	Source
bamUtil	1.0.15	https://genome.sph.umich.edu/wiki/BamUtil:_ bam2FastQ
MultiQC	1.13	https://github.com/ewels/MultiQC
Genomescope	2.0	https://github.com/tbenavi1/genomescope2.0
hifiasm	0.19.3	https://github.com/chhylp123/hifiasm
purge_dups	1.2.6	https://github.com/dfguan/purge_dups
BUSCO	5.3.2	https://busco.ezlab.org/
Merqury	1.3	https://github.com/marbl/merqury
Assembly-stats	17.02	https://github.com/rjchallis/assembly-stats
Arima-HiC Mapping Pipeline	-	https://github.com/ArimaGenomics/mapping_pipeline
YaHS	1.1	https://github.com/c-zhou/yahs
HiGlass	1.11.7	https://github.com/higlass/higlass
samtools	1.9	https://www.htslib.org/
PretextView	-	https://github.com/sanger-tol/PretextView/tree/master
BUSCO	5.7.0	https://busco.ezlab.org/
BlobToolKit	4.3.5	https://github.com/blobtoolkit/blobtoolkit
pbmm2	1.13.1	https://github.com/PacificBiosciences/pbmm2
Blast	2.15.0+	https://blast.ncbi.nlm.nih.gov/Blast.cgi

## Ethics and consent

 All work was conducted with approval by the AMNH Institutional Animal Care and Use Committee (AMNHIACUC-20191212).

## Data Availability

The *M. alvarezi* genome sequencing initiative is part of the Bat1K genome sequencing project. The genome assembly is released openly for reuse. The *M. alvarezi* genomic data is available on GenomeArk in this link: https://www.genomeark.org/bat1k-curated-assembly/Molossus_alvarezi.html Underlying data may be available for non-commercial research purposes upon request. Please email info@batbio.org for more information. The genome assembly can be found in the European Nucleotide Archive: Molossus alvarezi (Alvarez's mastiff bat). Accession number: GCA_037157525.1, https://www.ebi.ac.uk/ena/browser/view/GCA_037157525.1 ( BAT1K, 2024a) In the NCBI database, the BioProject for Molossus alvarezi isolate: mMolAlv1 (Alvarez's mastiff bat) is listed under Accession number: PRJNA944206, https://www.ncbi.nlm.nih.gov/bioproject/PRJNA944206. This project is part of the broader Bat1K BioProject PRJNA489245. ( BAT1K, 2024b) Data accession identifiers are reported in Table 1.
