# Supplementary material for: The genome sequence of Molossus alvarezi González-Ruiz, Ramírez-Pulido and Arroyo-Cabrales, 2011 (Chiroptera, Molossidae)
Source: Wellcome Open Res. 2024 Sep 12;9:522. [Version 1] doi: 10.12688/wellcomeopenres.22726.1 (PMC11474149; doi:10.12688/wellcomeopenres.22726.1)
Supplement: Peer review history

# Reviewer report: Reviewer response for version 1

**DOI:** 10.21956/wellcomeopenres.25029.r99870

**Published:** 2024-10-14

**Authors:** M. Alejandra Camacho

**Affiliations:** 1 Facultad de Ciencias Exactas y Naturales, Pontificia Universidad Catolica del Ecuador Escuela de Ciencias Biologicas, Quito, Pichincha, Ecuador

Dear Editor and Authors,

 The genome assembly presented in this manuscript provides bases for future studies on the species' evolutionary history, genetic diversity, and conservation status. The use of advanced sequencing technologies demonstrates a high level of technical rigor, and the fully phased assembly represents a key milestone for bat genomics. I believe that with some minor clarifications and adjustments, this work will make an important contribution to the field.

**Figure 1 (Phylogeny):** The figure, as presented, is not sufficiently informative. It does not accurately convey the phylogenetic position of *Molossus alvarezi* within Molossidae, as it lacks representation of other Molossus species. While the legend mentions that "within *Molossus*, *M. alvarezi* occupies a basal branch and is sister to a large clade of other species from Central and South America," this key phylogenetic relationship is neither shown nor easily inferred from the figure. Including additional *Molossus* species in the phylogeny would provide the necessary context to support the legend's claim and improve the overall clarity and scientific value of the figure.

**Genome Assembly (Table 1):** The genome assembly report provides a detailed overview of the sequencing and assembly process; however, there are a few discrepancies or missing details that should be addressed for clarity and consistency. For instance, the text mentions a total assembly length of 2.48 Gb in 504 scaffolds, while Table 1 indicates 187 scaffolds, potentially causing confusion for the reader. Additionally, the reported scaffold N50 in the text is 112.65 Mb, which differs from the value listed in Table 1 (113.92 Mb). Such differences, albeit small, should be reconciled.

**BUSCO Scores (Table 1):** The description of the BUSCO scores in the text mentions specific categories (C = complete, S = single copy, D = duplicated, F = fragmented, M = missing), but these are not reflected or explained in **Table 1** **.** Including a column or at least a footnote in the table that breaks down these BUSCO scores by category would significantly improve the clarity and informativeness of the table. As it stands, the reader is left without the specific breakdown that is crucial for understanding the quality of the assembly in terms of completeness and potential duplications or gaps. Additionally, it would be helpful to see the percentage values for these categories, as only a general percentage of completeness (98.2%) is mentioned in the text, but not in the table.

**Figure 3:** The Hi-C Contact Map is a valuable visualization, but it would benefit from additional context or explanation to assist readers in interpreting the data. Currently, the figure is missing guidance on how to read the contact frequencies across the chromosomes, and the **axes** are not labeled, which makes it more difficult to interpret. Maybe genomic coordinates or chromosome numbers or positions (in base pairs). Including this information would help readers understand which regions of the genome are involved in the interactions shown. It would be important to highlight key chromosomal regions or explaining what dense contact regions signify.

**Figure 4:** This figure could be improved by incorporating the detailed BUSCO score breakdown that was listed in Table 1. Including the categories (C = complete, S = single copy, D = duplicated, F = fragmented, M = missing) within the figure or its legend would provide more immediate insight into the quality and completeness of the genome assembly. This would make the figure more comprehensive by allowing readers to assess the assembly's quality directly from the visual data, without having to cross-reference Table 1.

**Figure 5:** The GC coverage plot is informative, but it might benefit from clearer labeling of the axes and a brief explanation of the significance of different GC content levels for the chromosomes or scaffolds.

Are sufficient details of methods and materials provided to allow replication by others?

Yes

Is the rationale for creating the dataset(s) clearly described?

Yes

Are the datasets clearly presented in a useable and accessible format?

Yes

Are the protocols appropriate and is the work technically sound?

Yes

Reviewer Expertise:

Diversity, systematics, and taxonomy of neotropical bats

I confirm that I have read this submission and believe that I have an appropriate level of expertise to confirm that it is of an acceptable scientific standard.

# Reviewer report: Reviewer response for version 1

**DOI:** 10.21956/wellcomeopenres.25029.r99868

**Published:** 2024-10-07

**Authors:** Diego A Caraballo

**Affiliations:** 1 Universidad de Buenos Aires and CONICET, Buenos Aires, Argentina

The manuscript submitted by Simmons et al. presents the genome assembly of a female *Molossus alvarezi*. Using a methodology that generates long reads, along with a technique to capture the spatial proximity between different regions of the genome, the authors produced a high-quality assembly totaling 2.48 Gb in length, with a mean depth of 51x, organized into 24 chromosomal pseudomolecules. The assembly is fully phased and exhibits a BUSCO completeness of 98.2% relative to the Laurasiatheria reference set. Together, these metrics confirm that it is a high-quality genome assembly. The manuscript is well-written, provides all necessary information regarding the methods and software employed, and makes the generated assembly readily available.

**Minor comment:**

 In the introduction and Figure 1, the authors state that there are 15 extant species of Molossus. However, according to Loureiro et al. (2020), there were initially 14 species. Two additional species described later, M. melini (Montani et al. 2021) and M. paranaensis (Chambi et al. 2024), bring the actual total to 16 species.

Are sufficient details of methods and materials provided to allow replication by others?

Yes

Is the rationale for creating the dataset(s) clearly described?

Yes

Are the datasets clearly presented in a useable and accessible format?

Yes

Are the protocols appropriate and is the work technically sound?

Yes

Reviewer Expertise:

Phylogenetics, Phylogenomics, Molecular Evolution, Viral Zoonoses, Bats, Rodents

I confirm that I have read this submission and believe that I have an appropriate level of expertise to confirm that it is of an acceptable scientific standard.

## References

[1] : Single nucleotide polymorphisms (SNPs) provide unprecedented resolution of species boundaries, phylogenetic relationships, and genetic diversity in the mastiff bats (Molossus). *Mol Phylogenet Evol* .2020;143: 10.1016/j.ympev.2019.106690 106690 10.1016/j.ympev.2019.106690 31760152

[2] : A new species ofMolossus (Chiroptera: Molossidae) from Argentina. *Journal of Mammalogy* .2021;102(5) : 10.1093/jmammal/gyab078 1426-1442 10.1093/jmammal/gyab078

[3] : Revisiting Molossus (Mammalia: Chiroptera: Molossidae) diversity: Exploring southern limits and revealing a novel species in Argentina. *Vertebrate Zoology* .2024;74: 10.3897/vz.74.e122822 397-416 10.3897/vz.74.e122822
